# Effects of Antioxidants and Vitamins on the Proliferation of Human Diploid Cells

**DOI:** 10.5195/cajgh.2013.112

**Published:** 2014-01-24

**Authors:** Gaziza Dаnlybaeva, Assel Isabekova, Zhansaya Akhmadeyeva

**Affiliations:** National Center for Biotechnology, Astana, Kazakhstan

**Keywords:** vitamins, antioxidants, human diploid cells

## Abstract

**Introduction:**

Microelements, essential nutrients that are needed in small amounts including minerals such as calcium, zinc, iron and other vitamins (A, B, C, and etc.), are macronutrients necessary for a healthy life.

The role of micronutrients in vivo is well known, and there are several publications that have examined the effects of micronutrients on genomic stability. Furthermore, a number of vitamins and microelements are substrates and/or cofactors in metabolic pathways, which regulate DNA synthesis and/or repair and gene expression.

A deficiency in such nutrients may result in disruption of genomic integrity and alterations in DNA methylation patterns, linking cellular nutrition with change in gene expression. For example, lack of vitamin C is known to cause increased DNA oxidation and chromosomal damage. Vitamin A, as well as other micronutrients, have a protective effect, whereas higher concentrations are associated with increased DNA damage.

Ubiquinone (coenzyme Q10) and dihydroquercetin are used in therapy as antioxidant compounds and electron carriers, which reduce lipid peroxidation of cell membranes. However, previous studies indicate that various ubiquinone analogs may cause a divergent effect on oxidative stress and oxidative phosphorylation.

The aim of our study was to investigate the effect of vitamins A and C, coenzyme Q10, and dihydroquercetin on the proliferative potential of cultured human embryonic diploid fibroblasts (M-22).

**Methods:**

In the first series of experiments, nontoxic concentrations of vitamins for the cells were identified using MTT assay.

**Results:**

Vitamins A and C, dihydroquercetin of 1μM, and coenzyme Q10 of 5μM were nontoxic for human skin fibroblasts. In the second series of experiments, cell cultivation was carried out with nontoxic concentrations. A vitamin C concentration of 1μM for 7 consecutive passages increased the proliferation index (PI) compared to the control. Thus, the average PI in the experiments was 2.3, whereas in the control, it was 1.7. Similar results were obtained when dihydroquercetin was added to the growth medium. However, further cultivation of cells in the presence of vitamin C decreased PI to 1.4, while the control value remained the same. Daily examination revealed no morphological changes in the cell culture, but the cell growth had slowed significantly. The use of vitamin A in a nontoxic concentration of 1 μM reduced PI to 0.7 in the first passage, so further culturing of human cells with vitamin A was stopped.

**Conclusion:**

Studies examining the effect of different combinations of microelements on the proliferation of human diploid cells and the expression of specific proteins in them are still being conducted.

